# Chemical Modification of Pactamycin Leads to New Compounds with Retained Antimicrobial Activity and Reduced Toxicity

**DOI:** 10.3390/molecules29174169

**Published:** 2024-09-03

**Authors:** Artemis Tsirogianni, Nikolina Ntinou, Konstantina Karampatsou, George Dinos, Georgia G. Kournoutou, Constantinos M. Athanassopoulos

**Affiliations:** 1 Synthetic Organic Chemistry Laboratory, Department of Chemistry, University of Patras, 26504 Patras, Greece; a.tsirogian@ac.upatras.gr (A.T.); up1068911@ac.upatras.gr (K.K.); 2 Department of Biochemistry, School of Medicine, University of Patras, 26504 Patras, Greece; dnikol@outlook.com (N.N.); dinosg@upatras.gr (G.D.)

**Keywords:** antibiotics, pactamycin, protein synthesis

## Abstract

Pactamycin (PCT), an antibiotic produced by *Streptomyces pactum*, is a five-membered ring aminocyclitol that is active against a variety of Gram-positive and Gram-negative microorganisms, as well as several animal tumor lines in culture and in vivo. Pactamycin targets the small ribosomal subunit and inhibits protein synthesis in bacteria, archaea, and eukaryotes, but due to its toxicity is used only as a tool for biochemical research. Prompted by the successful and well-established procedure for the derivatization of antibiotics, we modified pactamycin by tethering basic amino acids to the free primary amino group of the aminocyclitol ring. Specifically, lysine, ornithine, and histidine were conjugated via an amide bond, and the antimicrobial activity of the derivatives was evaluated both in vivo and in vitro. According to our results, their antimicrobial activity was maintained at almost equal levels, while their toxicity was reduced compared to the parent molecule. These findings suggest that the new pactamycin derivatives can be considered as promising pharmacophores for the development of new antimicrobials that are able to combat the dangerously increasing resistance of pathogens to antibiotics.

## 1. Introduction

**Figure 1:**
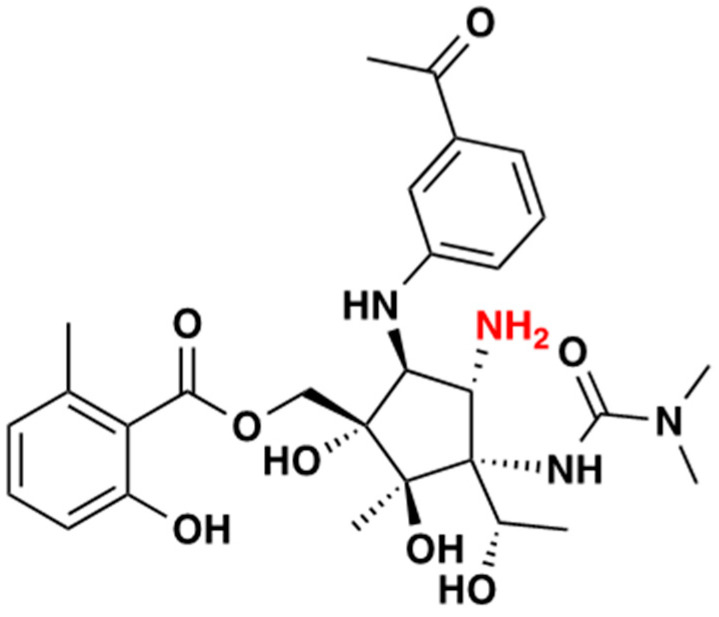
Pactamycin structure; the primary amine group (derivatization site) is shown in red.

Looking for new antibiotics to combat dangerously increasing pathogenic resistance, we decided to chemically modify the known antibiotic Pactamycin (PCT, Figure 1) in order to improve its biological profile. PCT is isolated from Gram-positive, filamentous soil bacteria *Streptomyces,* which are known for their ability to produce a wide variety of secondary metabolites with interesting pharmacological activities [1,2]. The molecule of PCT (Figure 1) bears a 5-membered aminocyclitol ring and, therefore, is structurally related to aminoglycoside antibiotics. After its discovery, it was initially thought to be a potential anticancer agent, but it was also found to have additional antiprotozoal and antiviral properties as well as potent action as an antimicrobial agent against both Gram-positive and Gram-negative bacteria [3,4,5]. It targets the E-site of the small ribosomal subunit in bacteria, archaea, and eukaryotes, where it strongly inhibits protein synthesis [6,7,8]. Initially, PCT was accepted as an inhibitor of translation initiation [9]. It was subsequently shown to be an inhibitor of translocation [7,8,10], a mode of action that agrees with both structural and biochemical experiments. Surprisingly, the inhibition of PCT on translocation was not general but dependent on the substrate that occupies the A-site, i.e., the characteristics of the pre-translocation complex have a significant influence on activity [7].

**Figure 2:**
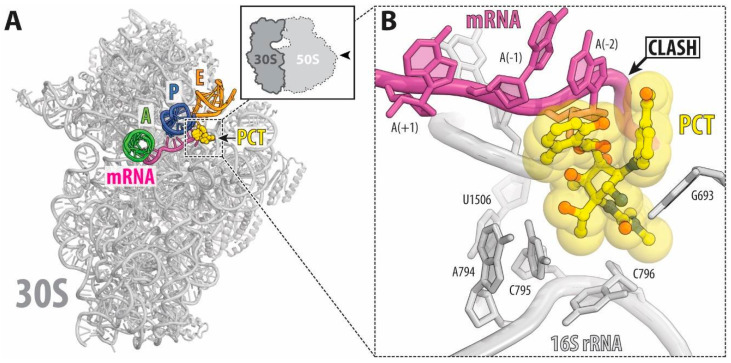
(**A**) Overview of the PCT binding site (yellow) on the *T. thermophilus*, co-crystallized with 70S ribosomes, mRNA (magenta), and tRNAs (green for the A site, dark blue for the P site and orange for the E site). (**B**) Binding of Pct to the ribosome displaces the mRNA from its normal location (Steric clashes are indicated by black arrow. *E. coli* numbering of the nucleotides in the 16S rRNA is used [8].

This PCT specificity has been confirmed by demonstrating that in vitro translation was arrested at preferential sites, which varied among the different mRNA templates used [11]. PCT could readily inhibit translocation of the ribosome carrying initiator tRNA^fMet^ in the P site and the peptidyl-tRNA mimic, N-Ac-Lys-tRNA^Lys^, or N-Ac-Val-tRNA^Val^ in the A site. However, when a similar complex was carrying deacylated initiator tRNA^fMet^ in the P site, with the A site occupied by N-Ac-Phe-tRNA^Phe^, no inhibition of translocation was observed, even at elevated concentrations of PCT [7]. The term “context-specific” refers to this property, which is shared by many other ribosome-acting antibiotics. This viewpoint differs from the idea that antibiotics are general inhibitors that arrest genes equally efficiently at every codon but inhibit differently based on mRNA sequencing and the substrates that are bound to the ribosome [12]. Even without considering the contribution of the ribosome-associated nascent proteins and mRNA structures, which change as the ribosome moves from codon to codon, the variety of ribosomal complexes potentially available in the cell to interact with an antibiotic is enormous. The effect of PCT at the genome-wide level and the molecular mechanisms responsible for the site-specificity of the drug action are yet to be investigated [13]. The binding site of PCT on the *T. thermophilus* ribosome has been determined by X-ray crystallography [6,8,14], and according to the data, PCT occupies the E-site of the 30S subunit and prevents translocation. This interaction is illustrated in Figure 2, showing the ribosomal complex of *T. thermophilus* with mRNA, tRNA_S_, and PCT [8]. The structure indicates that PCT stacks on nucleotide G693 of 16S rRNA and shifts mRNA out of its usual location in the E-site of the small subunit, thus disrupting mRNA’s interactions with the E-site tRNA.

Due to its toxicity, PCT ceased to be an attractive target for modification and is used now only as a tool to dissect translation. The complexity of the PCT molecule, with six contiguous stereocenters, a primary amine, and a urea moiety, plus the low efficiency of the currently used culture conditions, have generally kept the extent of PCT derivatization low [15]. However, the total chemical synthesis of PCT [16,17] and the discovery of its antiprotozoal activity [4] have reinvigorated the search for derivatives and resulted in many compounds with noticeable activities, especially against malaria parasites [17,18,19,20]. Since the derivatization of established pharmacophores is still the most efficient way to broaden the antibiotic pipeline, we have coupled the primary amine of the PCT cyclitol ring here with the basic amino acids histidine, lysine, and ornithine via an amide bond and assessed their efficacy both in vivo and in vitro. Based on previous studies, these basic amino acids maintained or enhanced the antibacterial action of the antibiotic chloramphenicol when they were coupled to it, either by substituting the dichloroacetyl moiety or tethering to the secondary hydroxyl group [21,22]. This successful modification strategy using basic amino acids prompted us to modify PCT using this approach. According to our results, the new compounds maintained almost equal antimicrobial activity while their toxicity was reduced compared to the parent molecule, suggesting that new pharmacophores are available with the potential to be further improved as antimicrobial agents.

## 2. Results

### 2.1. Chemical Synthesis

**Scheme 1:**
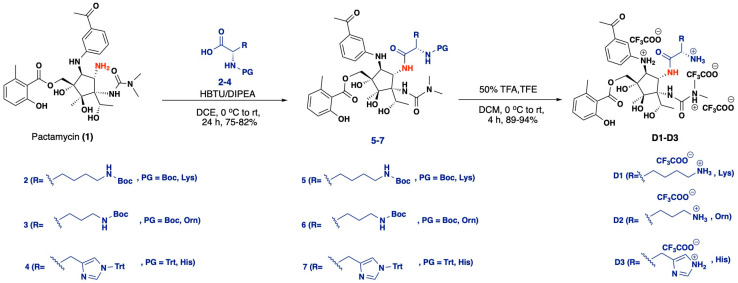
Synthetic procedure for intermediates 5–7, reagents and conditions: HBTU/DIPEA, DCE, 0 °C to RT, 24 h, 75–82%; and the final products D1-D3, reagents and conditions: 50% TFA, TFE, DCM, 0 °C to RT, 4 h, 89–94%.

The proposed PCT derivatives (Scheme 1) were synthesized from the readily available PCT **(1)** through coupling, using the system HBTU/DIPEA with Boc- or Trt-*N*-protected lysine, ornithine and histidine **(2–4)** respectively, which gave the corresponding derivatives **4–6** in 75–82% yield. TFA-mediated *N*-deprotection of the latter afforded the final Pct-amino acid conjugates **D1–D3** in 89–94% yield.

### 2.2. Antibacterial Activity

The new synthetic compounds were first tested in vivo. The MIC values were measured in both Gram-positive and Gram-negative pathogens. We used the *E. coli* K12 strain as the negative one and *S. epidermidis* as the positive one. According to Table 1, all of the tested compounds have kept their antimicrobial activity, although at a slightly lower level as compared to the mother molecule (PCT). This allows us to consider them as potential new antimicrobial agents.

**Figure 3:**
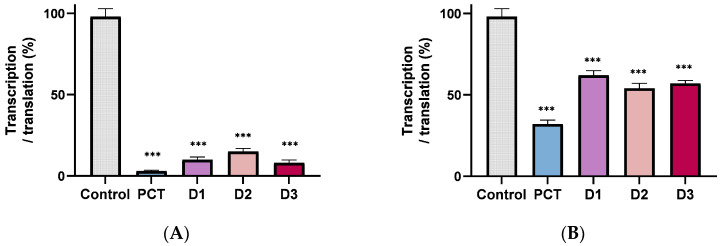
In vitro transcription translation. (**A**) in an *E. coli* system, and (**B**) in a reticulocyte lysate system. Antibiotic concentration is 10 μΜ. The product is expressed as a percent of control (without antibiotic). Asterisks indicate level of statistical significance: *** *p* ≤ 0.001.

Next, we explored their mechanism of inhibition in vitro to understand their mode of action further and catalog them as potential protein synthesis inhibitors. First, we used the coupled transcription-translation system, which resembles the real cellular conditions. In the assay, the T7 polymerase transcribes the *Renilla reniformis* luciferase gene and the protein yield, and the result of translation is approximated by monitoring the luminescence of the resulting luciferase protein [11,22]. As shown in Figure 3, the new derivatives are strong inhibitors of luciferase production, and taking into account that none of them inhibits transcriptionthe results clearly demonstrate that they inhibit ribosome function, although the exact step that is inhibited remains to be determined.

Inhibition in a eukaryotic system was investigated using the cell-free lysate of reticulocyte (Promega), keeping in mind that PCT also inhibits mammalian ribosomes and that its usage is discouraged due to inherent toxicity. According to Figure 3B, the inhibition of the derivatives is now lower than that of the parent compound, providing the first evidence that one can change the specificity and that the pactamycin derivatives have potential as lead compounds for developing new antimicrobial agents.

**Figure 4:**
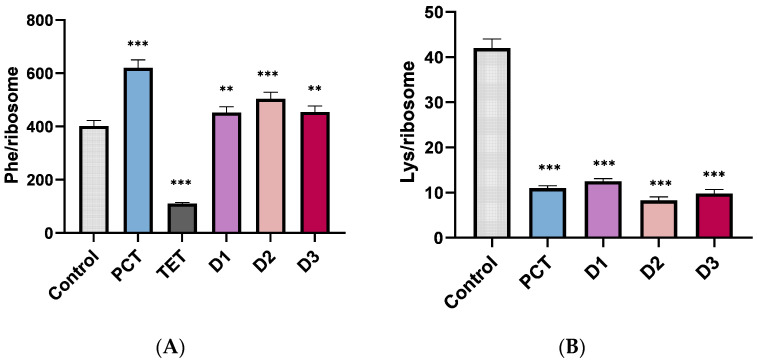
(**A**) Poly-phenylalanine and (**B**) Poly-lysine formation in the absence and presence of antibiotics. Each antibiotic was used at a concentration of 10 μΜ. Tetracycline (Tet, 10 μΜ) was used as a positive control. Asterisks indicate level of statistical significance: ** *p* ≤ 0.01, *** *p* ≤ 0.001.

The next in vitro assay was aimed at examining polymerization, namely poly-Phe and poly-Lys formation [7]. Ribosomes were programmed with poly (U) or poly (A), the responsible codons for phenylalanine and lysine incorporation, respectively. According to Figure 4A, poly-Phe formation is not inhibited, but poly-Lys (Figure 4B) is strongly inhibited. The findings in both situations are expected because it has been shown in earlier studies that PCT suppresses poly-Lys but not poly-Phe [7].

**Figure 5:**
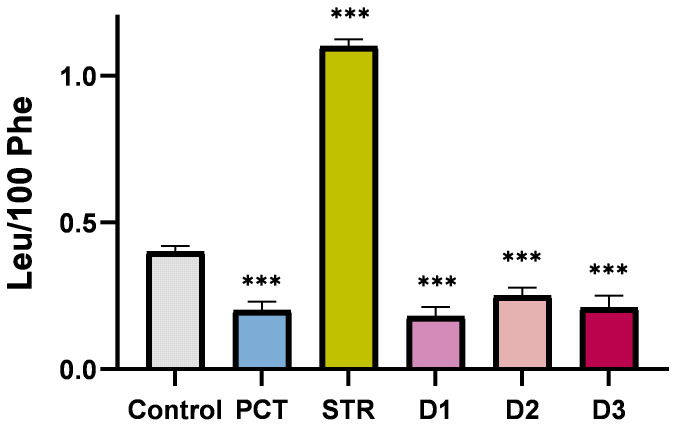
Misincorporation (Leu/100 Phe) in the absence and presence of 10 μΜ PCT and antibiotics. Streptomycin (STR) was used as a positive control for error coding. Asterisks indicate level of statistical significance: *** *p* ≤ 0.001.

To compare further the new compounds with the unmodified PCT, we employed a misincorporation assay to measure the incorporation near cognate amino acid leucine (UUC) at phenylalanine codons (UUU). It is known that many antibiotics occupying the small subunit interfere with mRNA decoding accuracy. While most of them had lower accuracy (streptomycin, paromomycin, and edeine), PCT, in contrast, was shown to increase accuracy [7,23,24]. According to Figure 5, all derivatives reduced the misincorporation at the level observed for PCT, thereby demonstrating a higher accuracy than the control. This evidence supports the suggestion that our derivatives share common characteristics with PCT and therefore might occupy the same binding site on the small subunit.

**Figure 6:**
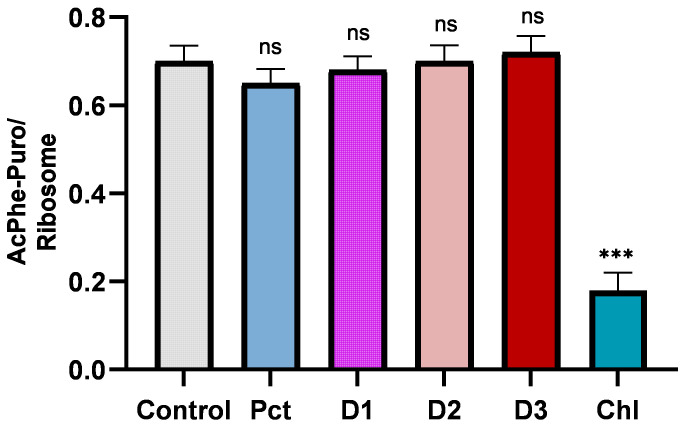
Puromycin reaction in the absence and presence of antibiotics at 10 μΜ concentration. Chloramphenicol (CHL) is used as a positive control that is known to occupy the A-site of the ribosomal large subunit and inhibit peptidyltransferase activity. Asterisks indicate level of statistical significance: *** *p* ≤ 0.001. ns: not significant changes.

Next, we tested the interference of our compounds with the peptidyltransferase center (PTC), which represents the main ribosomal enzymatic activity responsible for peptide bond formation and is localized in the central protuberance of the large subunit. The appropriate assay for such activity is the puromycin reaction since puromycin, as a pseudosubstrate, mimics the natural substrates, occupies the A-site, and favors peptide bond formation [25]. According to Figure 6, the system was not influenced by the presence of any of the pactamycin derivatives, suggesting that none of them interferes with the PTC, whereas the control antibiotic, chloramphenicol, which is known to bind at the PTC, was a strong inhibitor of the puromycin reaction [26]. Therefore, we suggest that, as in pactamycin itself, the binding site of the derivatives is far away from the PTC.

**Figure 7:**
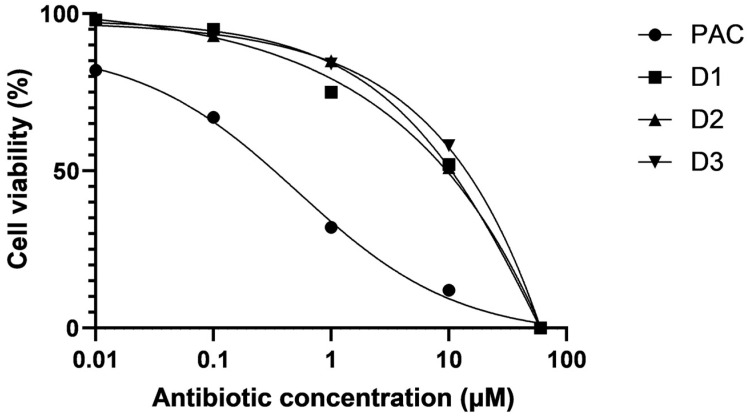
Resazurin assay. The compound’s toxicity was tested by measuring the viability of *HeLa* cells, after 16 h incubation with each antibiotic at various concentrations.

Lastly, we tested the toxicity of our compounds with resazurin assay, a well-established experimental approach to measure cell viability in the presence of alls compared to PCT. We used *HeLa* cell culture with exposure for 16 h to each of the compounds. Figure 7 indicates that the three derivatives were less toxic than PCT, with an IC_50_ 10-fold higher than that observed for pactamycin. This suggests that the derivatives are more advantageous for therapeutic application than PCT.

## 3. Discussion

Table 1 shows that the three PCT derivatives continue to be potent antibacterial agents with MIC values that are comparable to the parent molecule. Their antibacterial potency increases from D3 to D2 to D1 based on the MIC values, and they inhibit both Gram-positive and Gram-negative bacteria. As demonstrated by the in vitro transcription-translation assays (Figure 3), all compounds are potent inhibitors of protein synthesis in a system that closely mimics physiological circumstances. Additionally, a significant benefit for them as potential new therapeutic antibacterial agents is their decreased inhibitory activity in the eukaryotic reticulocyte lysate system as compared to PCT (Figure 3B).

The production of poly-Lys was prevented but not for poly-Phe. This is not surprising as pactamycin, the parent molecule, is known to inhibit poly-Lys but not poly-Phe [7]. Pactamycin more precisely blocks the translocation of the mRNA-tRNA_2_ complex by occupying the E-site of the small 30S subunit [8]. Furthermore, as was previously reported, this inhibition is context-specific; The AcVal-tRNA translocation was suppressed but not that of AcPhe-tRNA [12]. The three derivatives thus function as context-specific inhibitors and have a behavior similar to the parent molecule.

The puromycin reaction showed immunity in their presence. This is compatible with the functional step of the puromycin reaction, which does not include either codon recognition or translocation but only A-site occupation and spontaneous peptidyltransferase activity. This suggests that our derivatives occupy the small subunit E-site like PCT. The A-site of the large subunit remains free and the PTase activity remains totally active—being far away from the antibiotic binding site—since it is found in the center of the large subunit [27].

Furthermore, we tested the accuracy of the translation, since it is known that PCT increases the accuracy of amino acid incorporation in contrast to, for example, streptomycin, which is error-prone and increases the misincorporation level to nearly three times that of the control. As we can see in Figure 6, the three PCT derivatives increased the accuracy of decoding almost as much as PCT. This is an additional argument that the new compounds occupy the same binding site and function with the same inhibition mechanism.

Because PCT is so toxic, it has never been utilized as a therapeutic agent. Rather, a lot of work has been performed to reduce this negative side effect. Figure 7 shows that, although still hazardous, HeLa cell viability was higher in the presence of the new compounds than it was in the absence of them. Either way, these are positive facts that indicate what may be performed to minimize this negative impact.

Concerning the chemistry of modifications, it is important to mention that Boc-protection of the PCT primary amine group abolished almost all of its antibacterial activity Therefore, the participation of the basic amino acids in the new structures has a high impact on both the total activity and the side effects. This is in accordance with previous modifications of chloramphenicol with basic amino acids or polyamines either in the dichloroacetyl tail or in the primary hydroxy group [22,28,29]. Therefore, we suggest that the protonated amino groups of the basic amino acids are inserted into the negative charge relay of the ribosomal RNA scaffold to further interact, thus providing an additional nest of coulomb-type interactions. The described characteristics of our compounds may prove useful in the future since the balance between pathogen resistance and the development of new antimicrobial agents no longer exists as it did up to the end of the previous century. In the mid-1900s, when the antibiotic era first began, the majority of pathogenic microorganisms were more or less susceptible to all antibiotics [30,31]. However, natural selection-driven evolution has led to an increasing prevalence of resistance to all antibiotic medications, and the inability of the pharmaceutical industry to discover and develop new antibiotics is insufficient for the control of resistant pathogen infections [32].

Therefore, a variety of antibacterial agents that have been around for a while and have not been subjected to the natural pressure of resistance, due to their lack of therapeutic history, will prove beneficial in the future to battle the alarming rise in antibiotic resistance. One such potential drug is likely to be PCT, which, with the appropriate modifications, may mitigate undesirable side effects while remaining a safe antibacterial agent. As a result, our novel compounds offer a fresh scaffold that may be developed further into a potentially effective new antibacterial drug.

## 4. Materials and Methods

### 4.1. General Methods

^1^H NMR spectra were obtained at 600.13 MHz and ^13^C NMR spectra at 150.90 MHz on a Bruker AVANCEIII HD spectrometer. Chemical shifts (*δ*) are indicated in parts per million (ppm) downfield from TMS, and coupling constants (J) are reported in hertz (copies of ^1^H, ^13^C and HMBC NMR spectra are provided in the Supplementary Materials). ESI mass spectra were recorded at 30 V on amaZon SL ion trap mass spectrometer Bruker Daltonics using MeOH as solvent. High-resolution mass spectra (HRMS) were measured on a Bruker Maxis Impact QTOF Spectrometer. Anhydrous Na_2_SO_4_ was used for drying solutions, and the solvents were then routinely removed at ca. 40 °C under reduced pressure using a rotary vacuum evaporator. All reagents employed in the present work were commercially available at Merck KGaA, Darmstadt, Germany, and used without further purification. The protected amino acids 2–3 were purchased by BLD Pharmatech GmbH Reinbek Germany. Compound **4** was prepared as previously reported by us [22]. When required, reactions were carried out under dry argon atmosphere in preflamed glassware. Flash column chromatography (FCC) was performed on Merck silica gel 60 (230–400 mesh), and analytical thin layer chromatography (TLC) was performed on Merck silica gel 60F254 (0.2 mm) pre-coated on aluminum foil. Spots on the TLC plates were visualized with UV light at 254 nm using ninhydrin.

### 4.2. Experimental Procedures

#### 4.2.1. General Procedure for the Synthesis of Protected Pactamycin Derivatives

Pactamycin **(1)** was added (1.0 eq to an ice-cold solution of *N*-protected amino acids 2–4 (1.1 eq) in DCM (0.18 M), DIPEA (1.1 eq) and HBTU (1.1 eq). The reaction mixture was stirred at room temperature for 24 h, and the progress of the reaction was monitored by TLC. After completion of the reaction, the mixture was evaporated to dryness under vacuum, and the residue thus obtained was diluted with AcOEt and washed with 5% aqueous citric acid, H_2_O, and brine. The organic layer was dried over Na_2_SO_4_, filtered, and evaporated to dryness under vacuum. The residue was subjected to FCC to obtain the pure product as a pale-yellow oil (75–82%).
((1*S*,2*R*,3*R*,4*S*,5*S*)-5-((3-acetylphenyl)amino)-4-((*S*)-2,6-bis((tert-butoxycarbonyl)amino)hexanamido)-3-(3,3-dimethylureido)-1,2-dihydroxy-3-((*S*)-1-hydroxyethyl)-2-methylcyclopentyl)methyl 2-hydroxy-6-methylbenzoate (**5**): 79%; R_f_ (PhMe/AcOEt 4:6) 0.10; MS (ESI, 30 eV): m/z 888.5 [M + H]; ^1^H NMR (CDCl_3_) *δ* 7.39–7.36 (m, 1H), 7.23–7.18 (m, 3H), 6.83 (d, J = 7.6 Hz, 1H), 6.77 (d, J = 7.6 Hz, 1H), 6.59 (d, J = 6.7 Hz, 1H), 4.95 (d, J = 12.4 Hz, 1H), 4.77 (d, J = 12.4 Hz, 1H), 4.69 (s, 1H), 4.18 (s, 1H), 3.76–3.71 (m, 2H), 3.54–3.46 (m, 2H), 2.94 (s, 6H), 2.87 (s, 3H), 2.79 (s, 3H), 2.49 (s, 2H), 2.28 (s, 2H), 2.25–2.14 (m, 2H), 1.55 (s, 3H), 1.42 (s, 3H), 1.35 (d, J = 24 Hz, 18H); ^13^C NMR (CDCl_3_) *δ* 172.8, 158.8, 157.2, 137.9, 134.3, 129.3, 128.2, 126.4, 126.1, 122.9, 119.3, 118.5, 117.2, 115.5, 111.1, 71.3, 66.1, 64.4, 63.7, 54.2, 49.4, 49.3, 42.6, 40.3, 36.8, 35.8, 33.7, 30.5, 29.7, 28.4, 26.6, 25.5, 24.8, 20.9, 18.5, 13.7.
((1*S*,2*R*,3*R*,4*S*,5*S*)-5-((3-acetylphenyl)amino)-4-((*S*)-2,5-bis((tert-butoxycarbonyl)amino)pentanamido)-3-(3,3-dimethylureido)-1,2-dihydroxy-3-((*S*)-1-hydroxyethyl)-2-methylcyclopentyl)methyl 2-hydroxy-6-methylbenzoate (**6**): 75%; R_f_ (PhMe/AcOEt 3:7) 0.11; MS (ESI, 30 eV): m/z 896.5 [M + Na]; ^1^H NMR (CDCl_3_) *δ* 7.74 (d, J = 12.0 Hz, 1H) 7.65 (d, J = 6.0 Hz, 1H), 7.38–7.34 (m, 2H), 7.21–7.15 (m, 3H), 7.07 (br s, 1H), 6.81–6.74 (m, 2H), 6.56 (br s, 1H), 4.92 (d, J = 12.4 Hz, 1H), 4.77 (d, J = 12.4 Hz, 1H), 4.22–4.19 (m, 1H), 4.06 (t, J = 6.7 Hz, 1H), 3.78 (t, J = 5.8 Hz, 2H), 3.72 (s, 3H), 3.64 (t, J = 5.8 Hz, 2H), 3.06 (s, 1H), 2.94 (s, 6H), 2.48 (s, 2H), 1.54 (s, 3H), 1.41 (d, J = 13.7 Hz, 18H), 1.35 (d, J = 4.0 Hz, 6H); ^13^C NMR (CDCl_3_) *δ* 172.8, 158.8, 157.2, 137.9, 133.6, 129.3, 128.2, 126.4, 126.0, 122.9, 118.5, 117.2, 115.5, 111.1, 71.3, 63.7, 49.3, 42.6, 36.8, 33.7, 29.7, 28.4, 26.6, 25.5, 24.8, 23.3, 20.9, 18.5, 13.7.
((1*S*,2*R*,3*R*,4*S*,5*S*)-5-((3-acetylphenyl)amino)-3-(3,3-dimethylureido)-1,2-dihydroxy-3-((*S*)-1-hydroxyethyl)-2-methyl-4-((*S*)-3-(1-trityl-1H-imidazol-4-yl)-2-(tritylamino)propanamido)cyclopentyl)methyl 2-hydroxy-6-methylbenzoate (**7**): 82%; R_f_ (PhMe/AcOEt 7:3) 0.09; MS (ESI, 30 eV): m/z 1181.6 [M + H]; ^1^H NMR (CD_3_OD) *δ* 7.28–7.19 (m, 16H), 7.08–7.04 (m, 5H), 6.98–6.95 (m, 6H), 6.88–6.86 (m, 4H), 6.78–6.65 (m, 4H), 6.54–6.43 (m, 1H), 4.70–4.62 (m, 3H), 3.37–3.33 (m, 2H), 3.25 (s, 6H), 3.21 (s, 3H), 2.71 (s, 3H), 2.19 (s, 1H), 2.10 (d, J = 6.1 Hz, 2H), 1.45 (s, 6H); ^13^C NMR (CD_3_OD) *δ* 172.8, 158.8, 157.2, 150.2, 137.9, 134.2, 129.3, 128.2, 126.4, 126.0, 122.9, 118.5, 117.2, 115.5, 111.1, 71.3, 66.1, 64.4, 63.7, 49.3, 42.6, 36.8, 33.7, 29.7, 28.4, 26.6, 25.5, 24.8, 23.3, 20.9, 18.5, 13.7.

#### 4.2.2. General Procedure for the Deprotection of Pactamycin Derivatives

A solution of 50% TFA (3.0 eq) in DCM and TFE (2.0 eq) was added to an ice-cold solution of N-Boc or N-Trt-protected derivatives 5–7 (1.0 eq) in DCM (0.6 M). The reaction mixture was stirred for 30 min at 0 °C and then for 3.5 h at room temperature. Upon completion of the reaction, the mixture was triturated with Et_2_O and Hex to give the desired product as a yellow oil in 89–94% yield.

((1*S*,2*R*,3*R*,4*S*,5*S*)-5-((3-acetylphenyl)amino)-4-((*S*)-2,6-diaminohexanamido)-3-(3,3-dimethylureido)-1,2-dihydroxy-3-((*S*)-1-hydroxyethyl)-2-methylcyclopentyl)methyl 2-hydroxy-6-methylbenzoate (**D1**): 92%; R_f_ (DCM/MeOH/NH_3_: 9:1:0.1) 0.12; HR-ESI: m/z 687,3745; [M + H+] for the compound C_34_H_51_N_6_O_9_ requires 687.3712; ^1^H NMR (CD_3_OD) *δ* 7.20–7.15 (m, 3H), 7.07–7.04 (m, 2H), 6.67 (d, *J* = 6 Hz, 1H), 6.61 (d, *J* = 6 Hz, 1H), 5.13–5.09 (m, 3H), 4.83–4.79 (m, 3H), 4.46 (d, *J* = 12 Hz, 1H), 3.94–3.89 (m, 3H), 3.35–3.33 (m, 4H), 3.21–3.15 (m, 3H), 2.43 (s, 3H), 2.24 (s, 6H), 1.52 (s, 6H); ^13^C NMR (CD_3_OD) *δ* 202.3, 171.7, 150.1, 141.8, 139.5, 134.3, 131.0, 130.3, 129.6, 124.1, 120.6, 118.5, 116.1, 112.6, 84.2, 79.5, 74.6, 69.6, 68.2, 66.6, 60.4, 55.4, 42.0, 33.6, 27.6, 24.5, 23.7, 22.9, 18.8, 17.5, 15.3.
((1*S*,2*R*,3*R*,4*S*,5*S*)-5-((3-acetylphenyl)amino)-4-((*S*)-2,5-diaminopentanamido)-3-(3,3-dimethylureido)-1,2-dihydroxy-3-((*S*)-1-hydroxyethyl)-2-methylcyclopentyl)methyl 2-hydroxy-6-methylbenzoate (**D2**): 94%; R_f_ (DCM/MeOH/NH_3_: 9:1:0.1) 0.1; HR-ESI: m/z 673,3586; [M + H+] for the compound C_33_H_48_N_6_O_9_ requires 673,3556; ^1^H NMR (CD_3_OD) *δ* 7.12–7.09 (m, 2H), 7.01–6.98 (m, 2H), 6.63–6.56 (m, 3H), 4.73–4.70 (m, 2H), 3.29 (s, 6H), 3.13 (d, *J* = 6 Hz, 2H), 3.01–2.97 (m, 2H), 2.90–2.87 (m, 2H), 2.79 (s, 3H), 2.39 (s, 3H), 2.18 (d, *J* = 6 Hz, 4H), 1.79–1.76 (m, 4H), 1.39 (s, 6H); ^13^C NMR (CD_3_OD) *δ* 202.1, 174.3, 171.5, 141.6, 134.3, 131.0, 129.2, 124.1, 120.3, 119.4, 116.0, 89.5, 84.9, 83.7, 77.9, 75.2, 75.0, 66.6, 44.7, 39.7, 38.0, 33.5, 29.6, 27.6, 25.9, 24.5, 22.6, 18.3, 16.6, 15.2.
((1*S*,2*R*,3*R*,4*S*,5*S*)-5-((3-acetylphenyl)amino)-4-((*S*)-2-amino-3-(1H-imidazol-4-yl)propanamido)-3-(3,3-dimethylureido)-1,2-dihydroxy-3-((*S*)-1-hydroxyethyl)-2-methylcyclopentyl)methyl 2-hydroxy-6-methylbenzoate (**D3**): 89%; R_f_ (DCM/MeOH/NH_3_: 9:1:0.1) 0.09; HR-ESI: m/z 696,3376; [M + H+] for the compound C_34_H_45_N_7_O_9_ requires 696,3352; ^1^H NMR (CD_3_OD) *δ* 7.18–7.13 (m, 4H), 7.06–7.03 (m, 3H), 6.66 (d, *J* = 12 Hz, 1H), 6.59 (d, *J* = 12 Hz, 1H), 4.81–4.77 (m, 3H), 3.90–3.86 (m, 3H), 3.61 (s, 1H), 3.36 (s, 3H), 3.32 (s, 2H), 2.41 (s, 6H), 2.23 (s, 3H), 1.5 (s, 6H); ^13^C NMR (CD_3_OD) *δ* 202.0, 167.1, 130.6, 130.1, 129.4, 128.8, 119.4, 114.8, 113.7, 110.8, 106.3, 96.0, 80.2, 70.3, 68.9, 66.1, 56.7, 44.6, 35.6, 31.5, 30.0, 27.5, 26.8, 20.9, 19.6, 18.8, 18.2.

### 4.3. Materials

L-[^3^H]-phenylalanine, L-[^14^C]-phenylalanine, L-[^3^H]-leucine, L-[^3^H]-lysine, and L-[^3^H]-Valine were obtained from Moravek Inc., Quality Radiochemicals and Labeled compounds (Brea, CA, USA). Filter count scintillation liquid was purchased from Perkin Elmer (Richmond, CA, USA). tRNA bulk, ATP, poly (A), and poly (U) were from Merck (Kenilworth, NJ, USA). Pactamycin was kindly provided by Professor C. Coutsogeorgopoulos. The resazurin powder was obtained from Sigma-Aldrich (St. Louis, MO, USA).

### 4.4. Bacterial Strains

The wild-type *E. coli* K12/MG1655 strain was used both to test the antimicrobial activity and to isolate the components of the functional assays such as ribosomes, tRNA synthetases, elongation factors, etc. [11]. *S. epidermidis* cells were grown in LB medium as was previously described [33].

### 4.5. Biochemical Preparations

Reassociated 70S ribosomes were prepared from *Escherichia coli* K12 cells as described previously [34] and kept in a buffer containing 20 mM HEPES/KOH (pH 7.6), 50 mM CH_3_COONH_4_, 6 mM (CH_3_COO)_2_Mg, and 4 mM β-mercaptoethanol. The S-100 fraction was prepared as previously described [35] and was treated with DE-52 cellulose to remove tRNAs and RNases. Ac [^3^H] Phe-tRNA and Ac [^3^H] Val-tRNA were prepared using specific tRNA under standard conditions [35] and were separated from uncharged tRNA by reverse-phase HPLC on a Nucleosil column using a programmed binary gradient of solvents 1 (20 mM ammonium acetate, pH 5.0, 10 mM magnesium acetate, 400 mM NaCl) and 2 (60% *v*/*v* methanol in buffer 1). The 70S ribosome-Poly (U)-Ac [^3^H] Phe-tRNA] complex, was prepared as described previously [7].

### 4.6. MIC

The microdilution method was used to determine the minimum inhibitory concentration (MIC) of our compounds that inhibits the visible growth of *E. coli* K12 strain. The MIC values were determined in triplicate by a liquid broth microdilution assay ([36]). Cells were grown overnight in Luria–Bertani medium and were diluted into fresh Luria–Bertani medium again and were grown. Early exponential-phase cultures were then diluted to a final absorbance at 0.050 (OD_600_) and then incubated with the appropriate antibiotic at 37 °C for 18 h with shaking. The MIC represents the lowest concentration of the drug that caused culture growth arrest.

### 4.7. Inhibition of Translation Using In Vitro Cell-Free Expression System

In this study, the S30 T7 high-yield protein expression system (Promega Corporation, Madison, WI, USA) was used. Screening assays of our compounds were performed in small-scale in vitro transcription/translation reactions using an internal control DNA template containing the *Renilla reniformis* luciferase gene as previously described [22]. From each reaction, 2.5 μL samples were taken and diluted by adding 97.5 μL of the lysis buffer from the *Renilla* Luciferase Assay kit (Biotium, Fremont, CA, USA) used. The mix was thoroughly mixed, and 50 μL was then placed into a 96-well white opaque plate (Greiner, Kremsmünster, Austria). Immediately before the measurement, 50 μL of the assay reagent was added to each sample, mixed, and immediately placed in a luminometer (Perkin Elmer Victor2) for measurement. Data were presented as percent of control (without antibiotic). Reticulocyte lysate was used for eukaryotic ribosome inhibition (Promega Corporation, Madison, WI, USA), and manufacturer instructions were followed.

### 4.8. Poly Phenylalanine Synthesis

The assay was carried out in a buffer containing 20 mM HEPES-KOH (pH 7.6), 150 mM CH_3_COONH_4_, 4.5 mM (CH_3_COO)_2_Mg, 4 mM β-mercaptoethanol, 2 mM spermidine, and 0.05 mM spermine. In the first incubation, reassociated 70S ribosomes (0.5 μM) were preincubated for 10 min at 37 °C with each antibiotic at the appropriate concentration. Then in a total volume of 15 μL, 25 μg of poly (U) mRNA was mixed with [^3^H] phenylalanine (5 nmol, 50 dpm/pmol), 1 *A*_260_ unit tRNA bulk (*E. coli*), 3 mM ATP, 1.5 mM GTP, 5 mM acetyl phosphate, and an optimized amount of S-100 fraction to maximize the picomole of phenylalanine polymerized per picomole of 70S ribosomes. After 30 min of incubation at 37 °C, hot trichloroacetic acid (TCA) precipitation was performed, and polypeptides were passed through glass fiber filters [36]. The radioactivity retained on the filters was measured in a liquid scintillation counter. The Phe/ribosome ratio represents the picomole of Phe incorporated into the polypeptide chain per picomole of 70S ribosomes as a function of time [7].

### 4.9. Poly Lysine

This assay was performed as described for poly (U)-dependent poly (Phe) synthesis, except that poly (A) was used instead of poly (U) and [^3^H] lysine (3 nmol, 500 dpm/pmol) was the amino acid source. The incubation was performed at 37 °C for 1 h, and the TCA solution used for precipitating poly-lysine peptides was treated with 0.25% sodium wolframate before use [7].

### 4.10. Misincorporation

Poly (U)-dependent poly (Phe) was performed as above except for the addition of 100 pmoles [^3^H] leucine (3000 dpm/pmol) and 200 pmol tRNA bulk. After incubation and hot TCA precipitation, the radioactivity resulting from [^14^C] phenylalanine and [^3^H] leucine isotopes could be accurately separated by scintillation counting. Misincorporation is expressed in terms of leucine molecules incorporated per 100 molecules of phenylalanine polymerized [7].

### 4.11. Puromycin Reaction

The reaction between the previously prepared post-translocation ribosomal complexes and an excess of puromycin in buffer A was carried out at 37 °C for 2 min [25]. The ionic conditions were 20 mM HEPES-KOH (pH 7.6), 4.5 mM magnesium acetate, 150 mM ammonium acetate, 2 mM spermidine, 0.05 mM spermine, and 4 mM β-mercaptoethanol, which were kept constant throughout all the steps of complex formation and puromycin reaction. This buffer approximates physiological conditions with respect to the concentrations of the essential ions using NH^4+^ ions instead of K^+^ [34].

The reaction volume was 20 μL with a final puromycin concentration of 1 mM in the absence or presence of 10 μM of each antibiotic. The reaction was stopped by the addition of an equal volume of 0.3 M sodium acetate saturated with MgSO_4_ at a pH of 5.5, with the resulting volume extracted using 1 mL of ethyl acetate and the radioactivity contained in 700 μL of the organic phase quantified by liquid scintillation. The results were expressed as the pmol ratio of Acetyl-[^3^H] phenylalanine-puromycin per pmol of ribosome.

### 4.12. Resazurin Assay

The *HeLa* cell lines were cultured in Dulbecco’s modified Eagle’s medium (DMEM), containing 10% (*v*/*v*) fetal bovine serum (FBS), 2 mM L-glutamine, 100 U/mL of penicillin and 100 U/mL of streptomycin (all from Gibco, Paisley, UK) in plastic disposable tissue culture flasks at 37 °C in 5% CO_2_. Five thousand cells (HeLa) were seeded in 96-well plates in DMEM/10% FBS and were treated with antibiotics at 10 μM concentration for 16 h at 37 °C. To provide the necessary control samples, every plate also included wells without cells but with an equal amount of medium as the other wells on the plate (blank control).

A resazurin solution (0.125 mg/mL) was prepared in water and filter-sterilized using a 0.22-µm membrane. The prepared solution was covered to prevent exposure to light and was stored at 4 °C [37]. Cells were then incubated for 2 h with commercial resazurin solution (Biotium) at 37 °C followed by absorbance-based detection (570 nm and 600 nm) according to the manufacturer’s instructions. Experiments were performed in triplicates.

## 5. Conclusions

Significant antibacterial activity was shown by the PCT derivatives when the primary amino group of the cyclitol ring was tethered with the basic amino acids lysine, ornithine, and histidine. These derivatives, like the parent chemical formula, effectively suppress the growth in both Gram-positive and Gram-negative bacteria and strongly inhibit protein synthesis in different cell-free systems. Compared to PCT, they are less toxic in a eukaryotic system, which is a remarkable benefit. Due to their ability to balance efficacy and adverse effects, the novel compounds of PCT show promise for development as safer antibacterial medicines and may be attractive candidates to be developed as new antimicrobials to combat antibiotic resistance in the future.

## Figures and Tables

**Table 1:** In vivo antibacterial activity. The microdilution method was used to determine the minimum inhibitory concentration (MIC) of our compounds that inhibits the visible growth of *E. coli* K12 strain or *S. epidermidis* after overnight incubation at 35 °C.

Compound	MIC (μM)		MIC (μgr/mL)	
	*E. coli*	*S. epidermidis*	*E. coli*	*S. epidermidis*
PCT	8	4	4.47	2.23
D1, PCT-Lysine	16	8	18.29	9.14
D2, PCT-Ornithine	16	8	18.08	9.04
D3, PCT-Histidine	32	16	36.86	18.43

## Data Availability

Data are contained within the article and Supplementary Materials.

## Supplementary Materials

The following supporting information can be downloaded at: https://www.mdpi.com/article/10.3390/molecules29174169/s1, Figures S1–S17: Copies of ^1^H, ^13^C and HMBC-NMR spectra of compounds **5–7** and **D1–D3**.
