# Supplementary material for: Chemical Modification of Pactamycin Leads to New Compounds with Retained Antimicrobial Activity and Reduced Toxicity
Source: Molecules. 2024 Sep 3;29(17):4169. doi: 10.3390/molecules29174169 (PMC11397182; doi:10.3390/molecules29174169)
Supplement: Supplementary file 1 [file molecules-29-04169-s001.zip › molecules-3151424-supplementary.pdf]

SUPPORTING INFORMATION

Chemical Modification of Pactamycin leads to new compounds that retain antimicrobial activity with reduced toxicity

Tsirogianni A.[1], Ntinou N.[2], Karampatsou K.[1], Dinos G.[2], Kournoutou G.G.[2,*] and Athanassopoulos C.M.[1,*]

1 Synthetic Organic Chemistry Laboratory, Department of Chemistry, University of Patras, GR-26504 Patras, Greece; a.tsirogian@ac.upatras.gr; kath@upatras.gr

2 Department of Biochemistry, School of Medicine, University of Patras, 26504 Patras, Greece; dnikol@outlook.com; dinosg@upatras.gr; gkurnutu@upatras.gr.

* Correspondence: gkurnutu@upatras.gr; kath@upatras.gr Tel.: 00302610-969125; 00302610997909

Copies of NMR spectra

Table of Contents

Figure S1. ^{1}H-NMR spectrum of compound 5 3

Figure S2. ^{13}C-NMR spectrum of compound 5 3

Figure S3. HMBC-NMR spectrum of compound 5 4

Figure S4. ^{1}H-NMR spectrum of compound 6 5

Figure S5. ^{13}C-NMR spectrum of compound 6 5

Figure S6. HMBC-NMR spectrum of compound 6 6

Figure S7. ^{1}H-NMR spectrum of compound 9 7

Figure S8. ^{13}C-NMR spectrum of compound 9 7

Figure S9. HMBC-NMR spectrum of compound 7 8

Figure S10. ^{1}H-NMR spectrum of compound D1 9

Figure S11. ^{13}C-NMR spectrum of compound D1 9

Figure S12. HMBC-NMR spectrum of compound D1 10

Figure S13. ^{1}H-NMR spectrum of compound D2 11

Figure S14. ^{13}C-NMR spectrum of compound D2 11

Figure S15. HMBC-NMR spectrum of compound D2 12

Figure S16. ^{1}H-NMR spectrum of compound D3 13

Figure S17. ^{13}C-NMR spectrum of compound D3 13

Figure S18. HMBC-NMR spectrum of compound D3 14

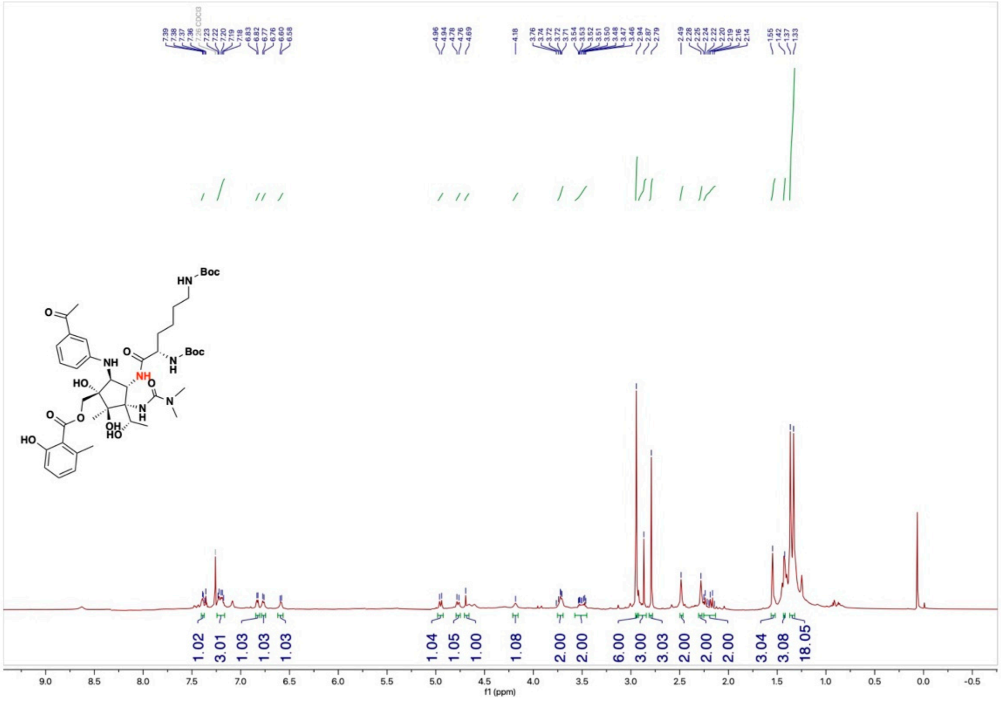

Figure S1. ^{1}H-NMR spectrum of compound 5

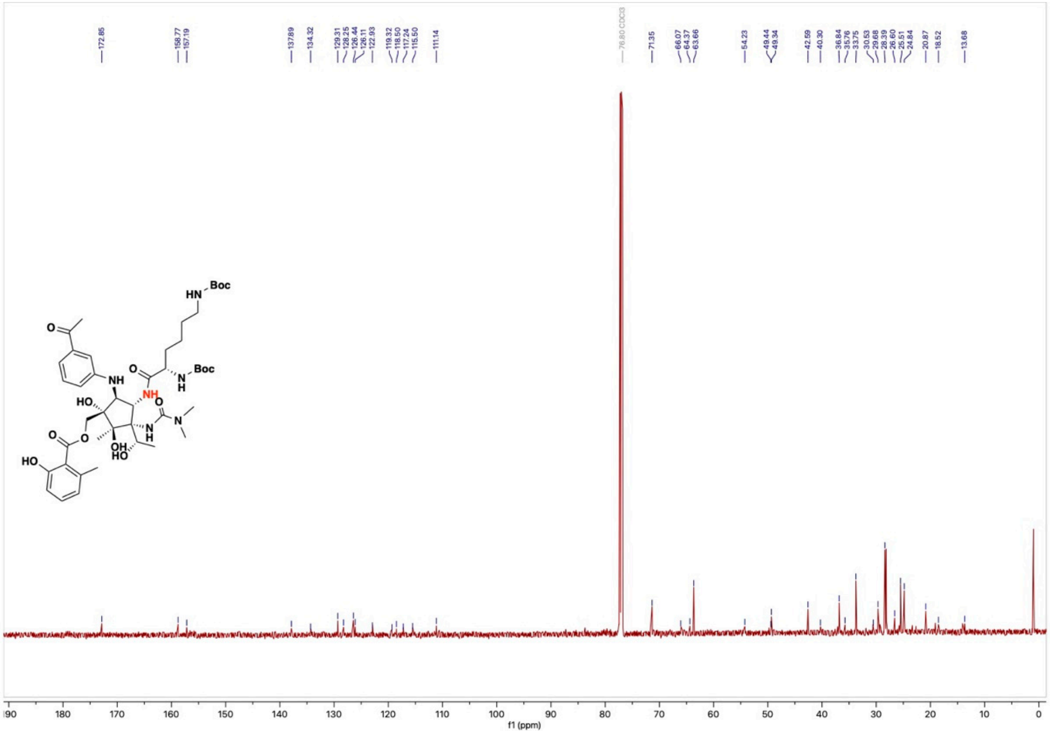

Figure S2. ^{13}C-NMR spectrum of compound 5

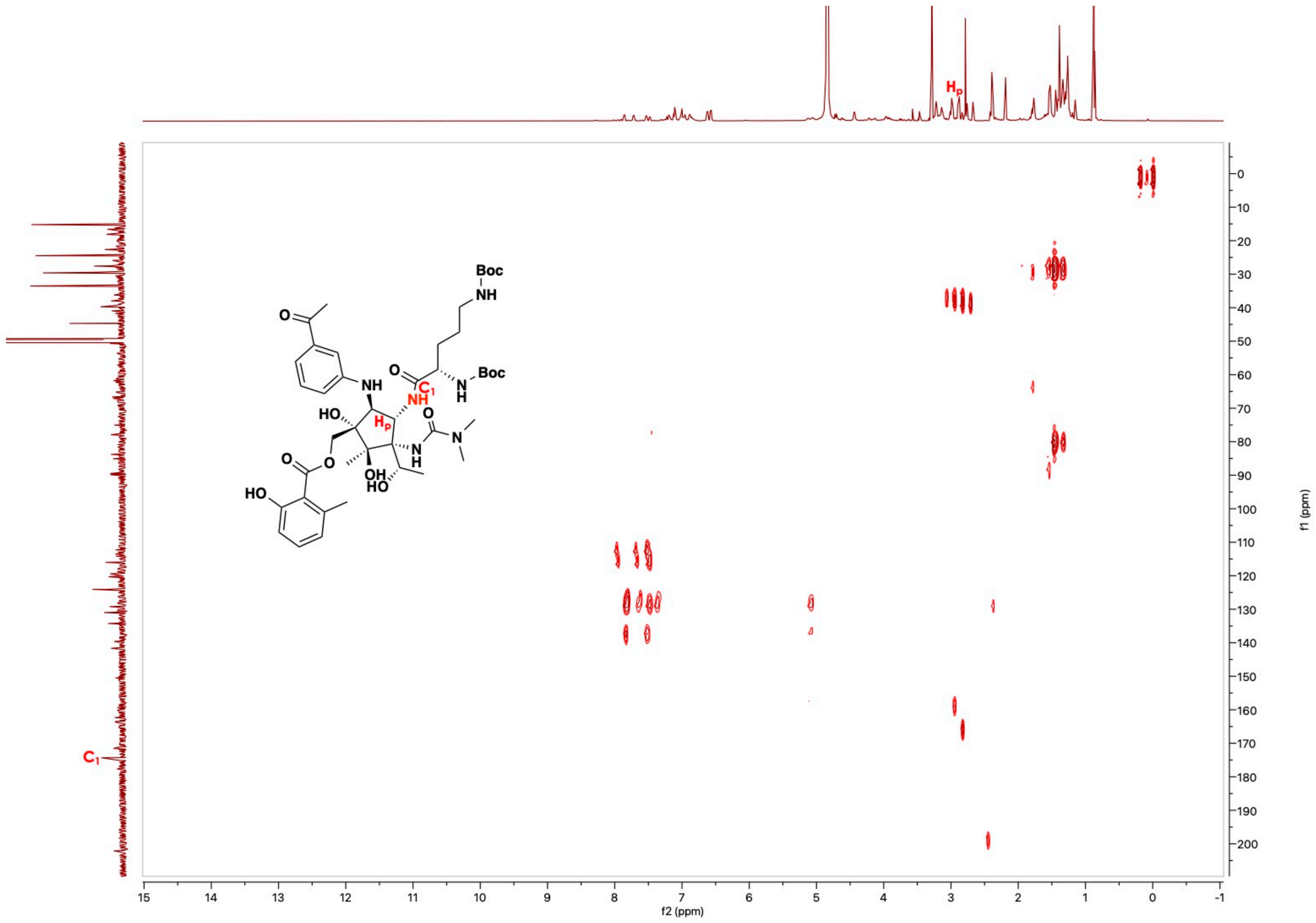

Figure S3. HMBC-NMR spectrum of compound 5

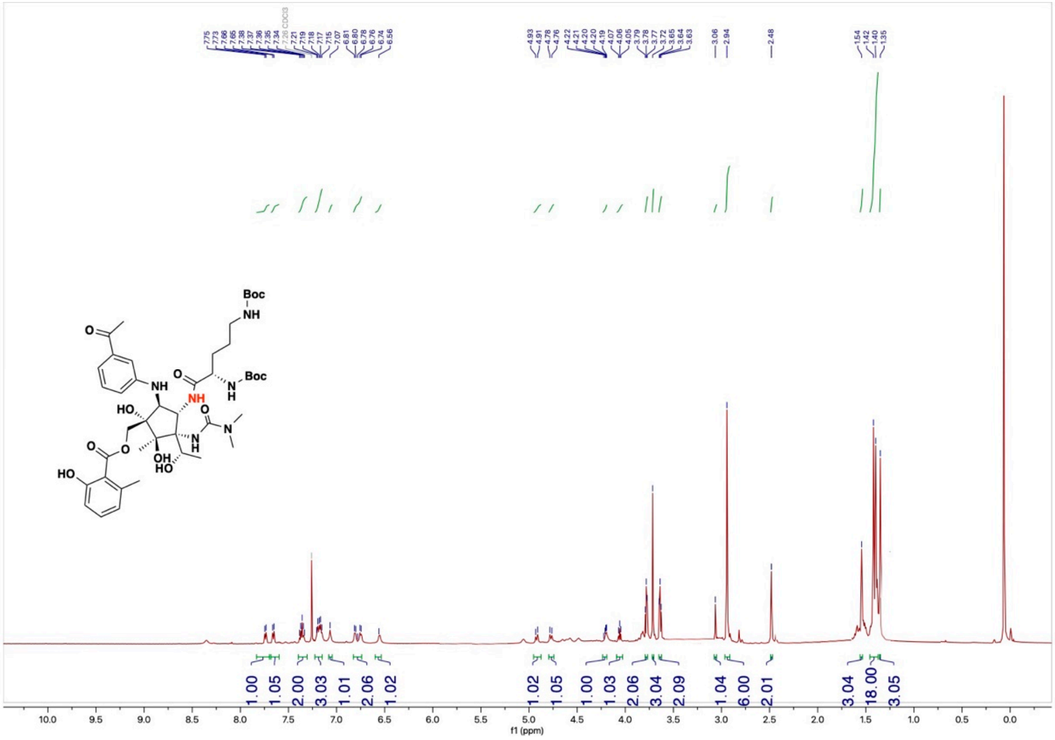

Figure S4. ^{1}H-NMR spectrum of compound 6

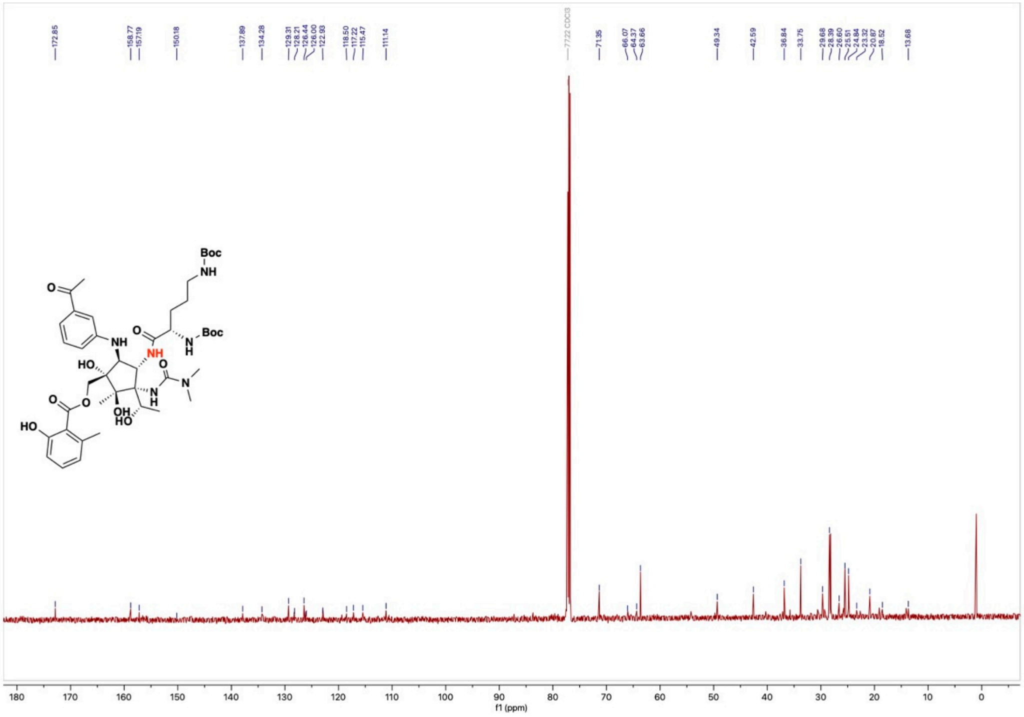

Figure S5. ^{13}C-NMR spectrum of compound 6

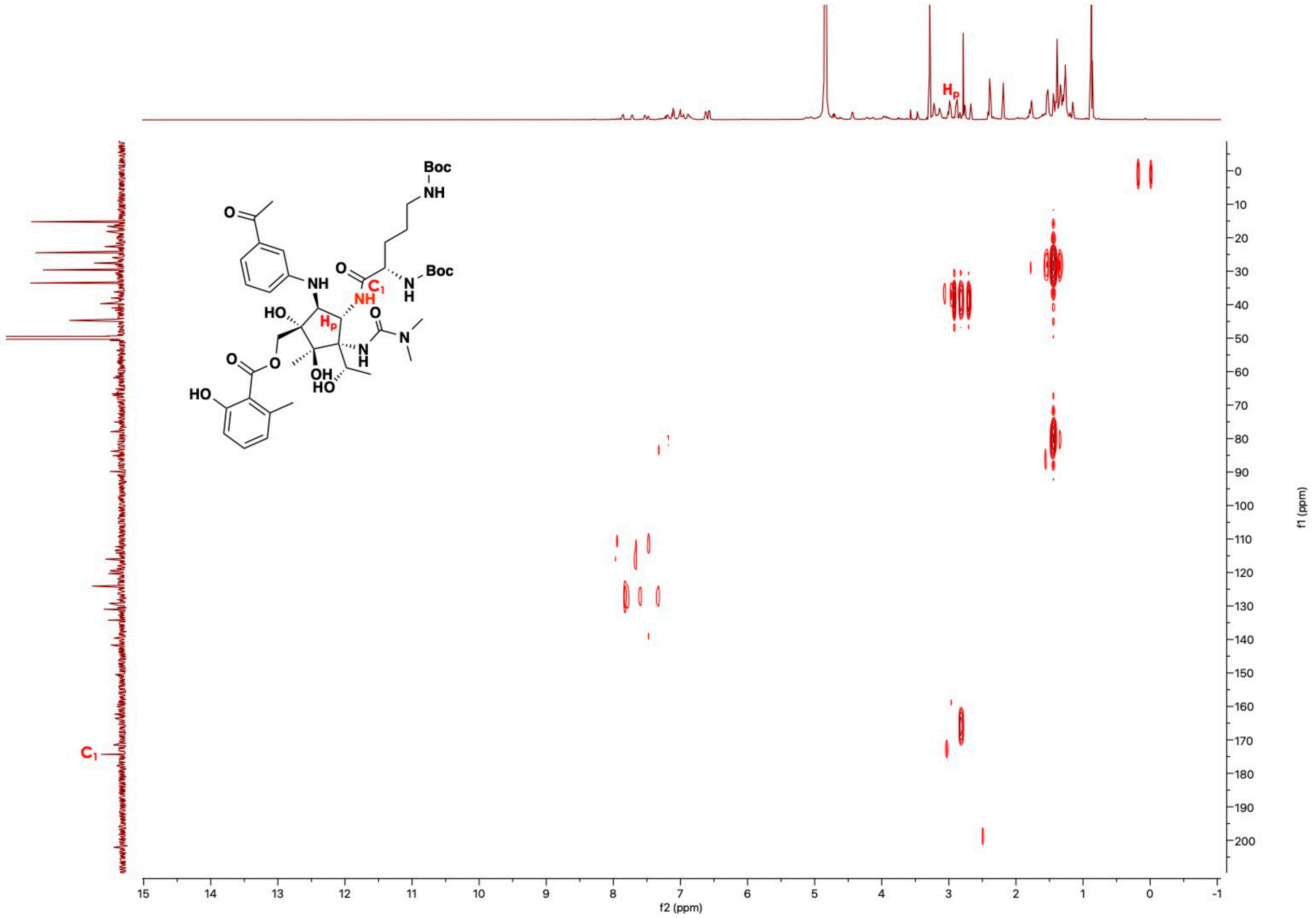

Figure S6. HMBC-NMR spectrum of compound 6

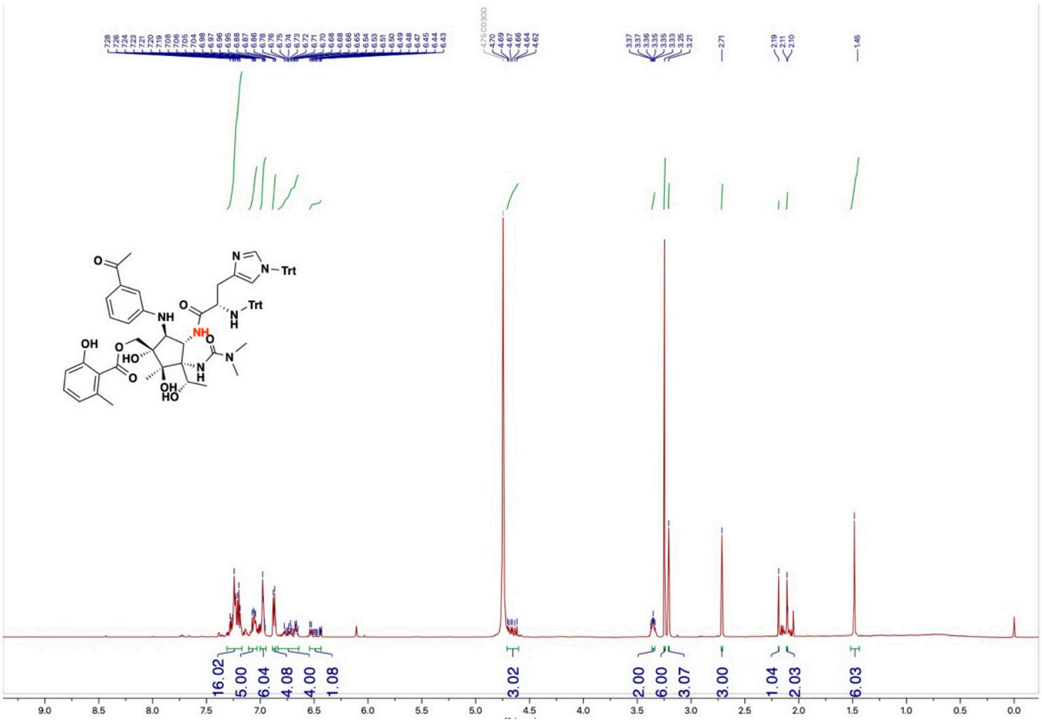

Figure S7. ^{1}H-NMR spectrum of compound 9

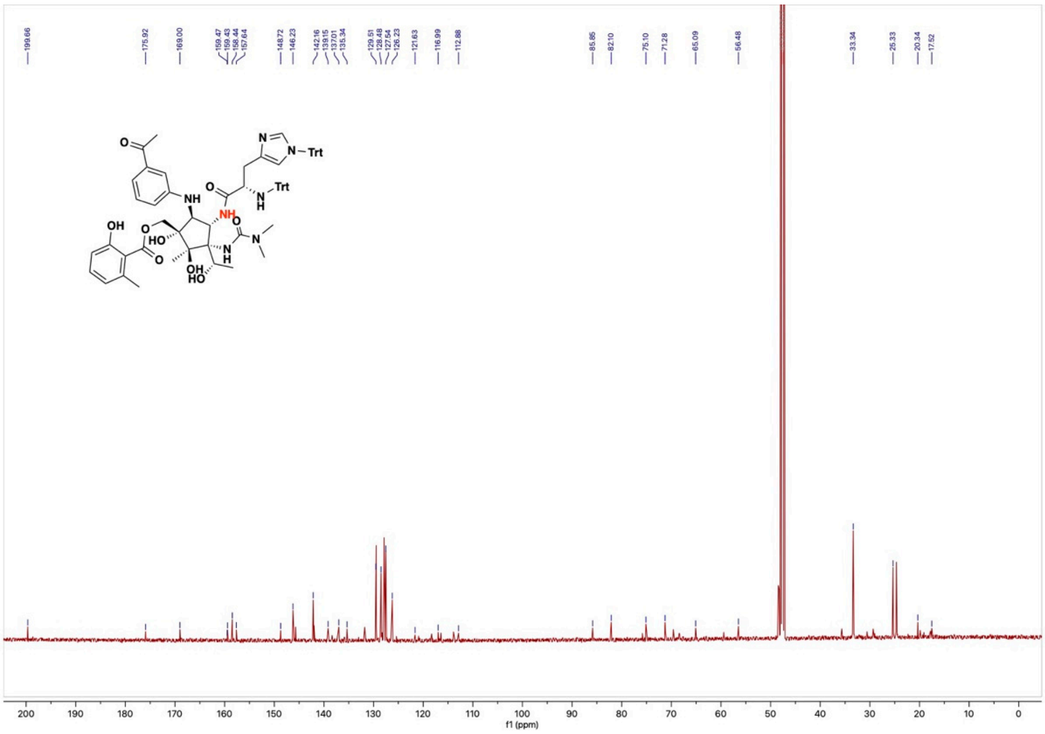

Figure S8. ^{13}C-NMR spectrum of compound 9

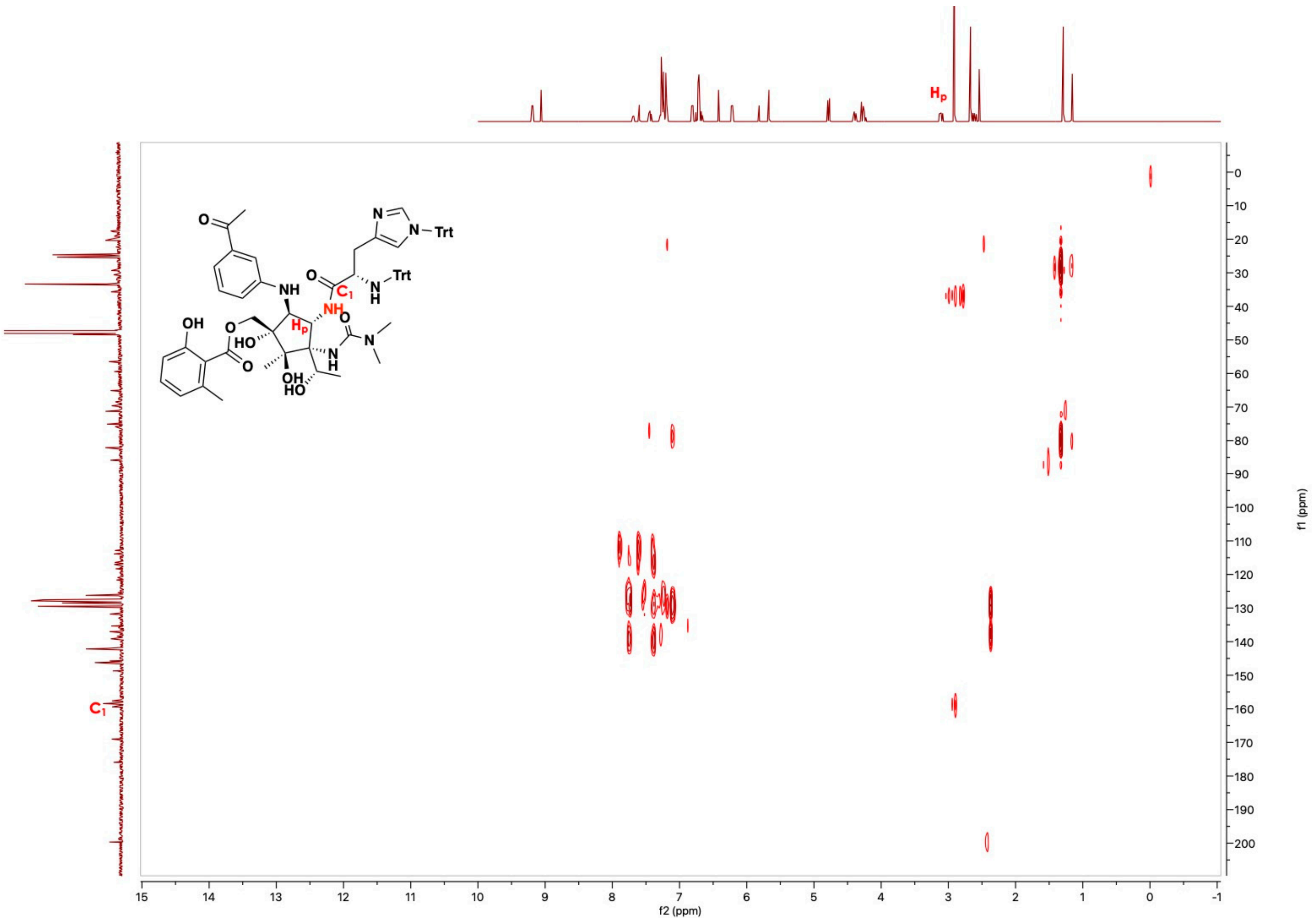

Figure S9. HMBC-NMR spectrum of compound 7

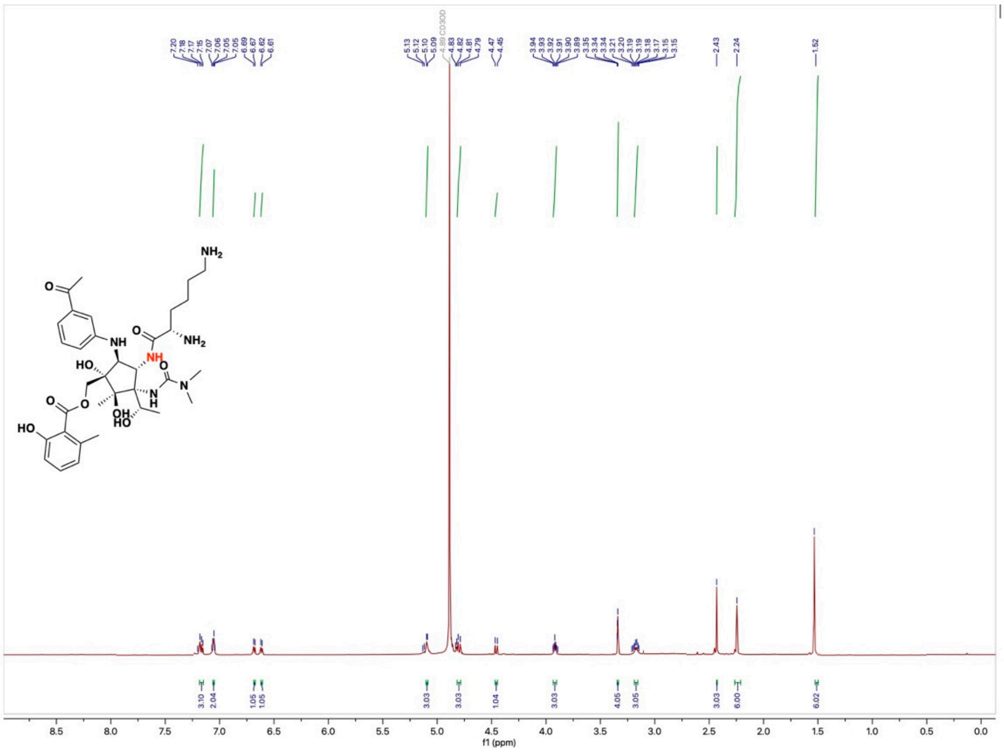

Figure S10. ^{1}H-NMR spectrum of compound D1

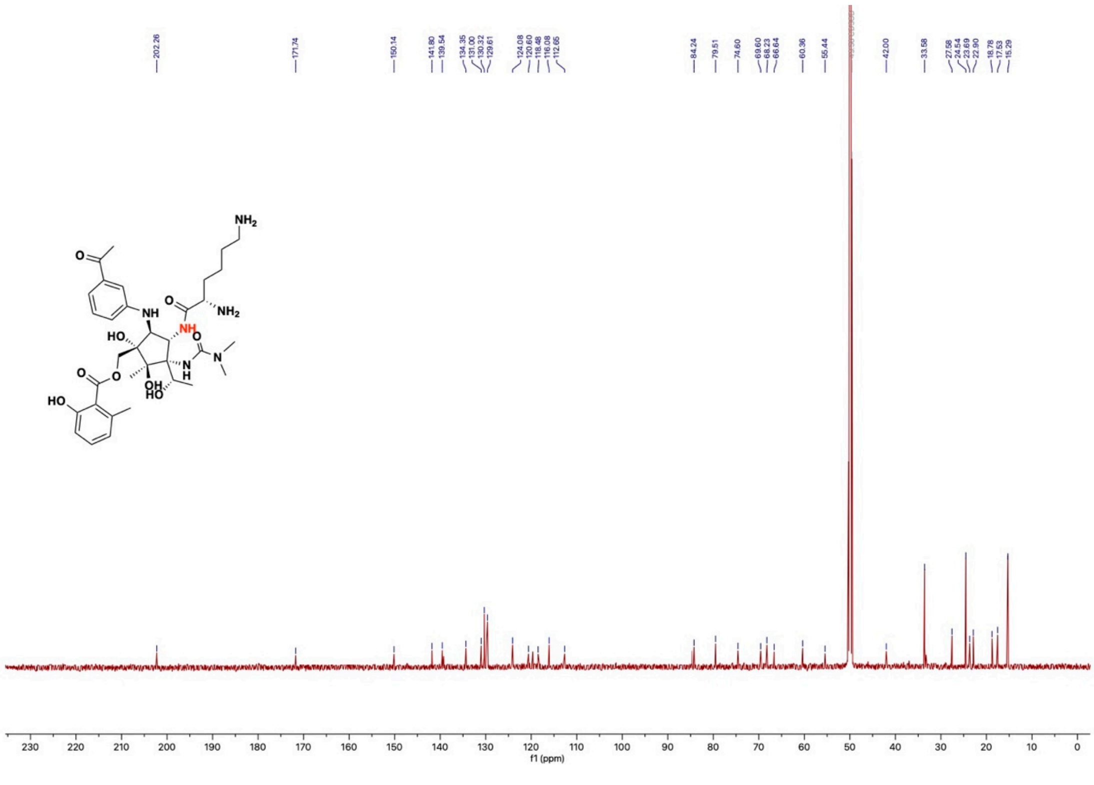

Figure S11. ^{13}C-NMR spectrum of compound D1

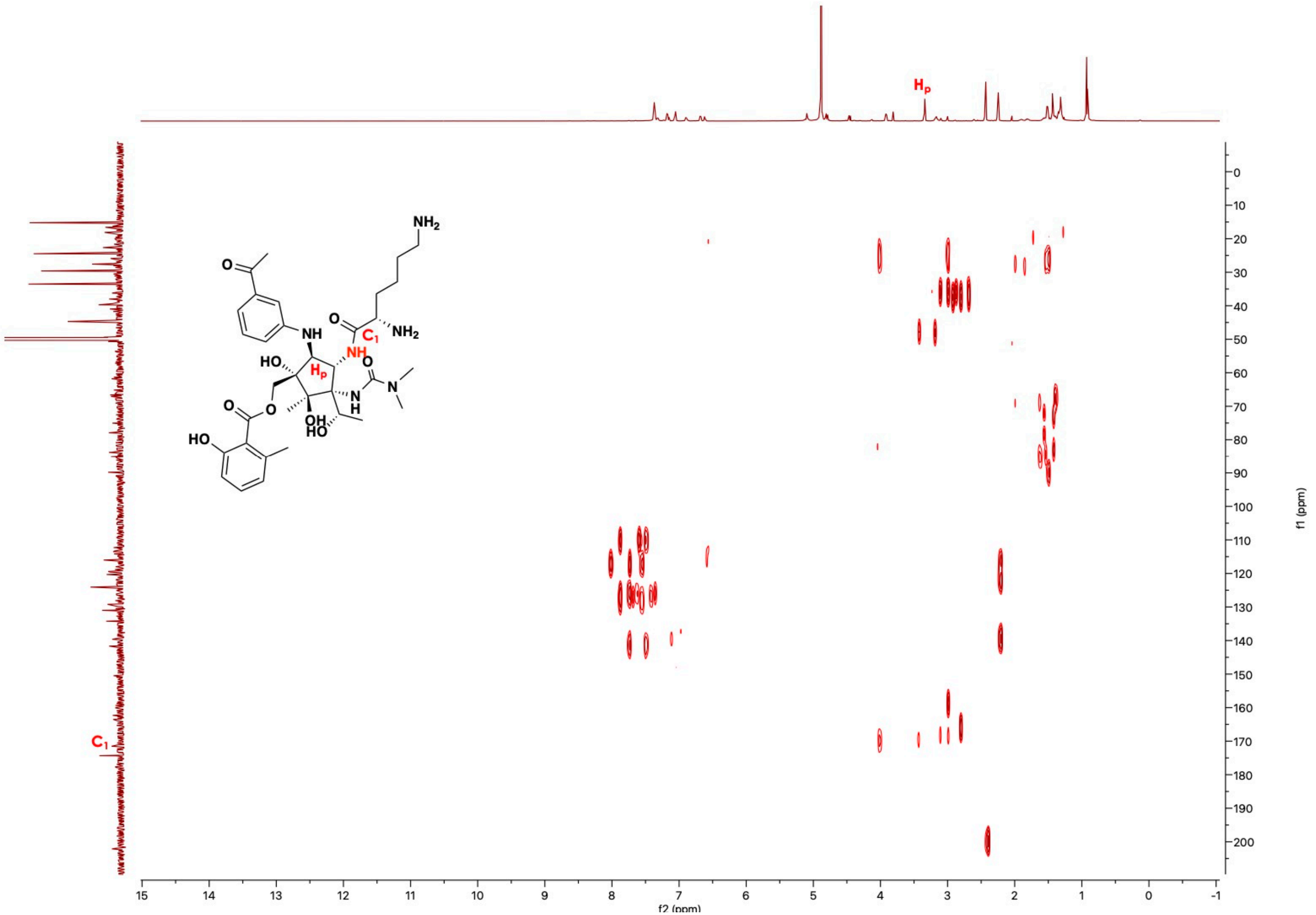

Figure S12. HMBC-NMR spectrum of compound D1

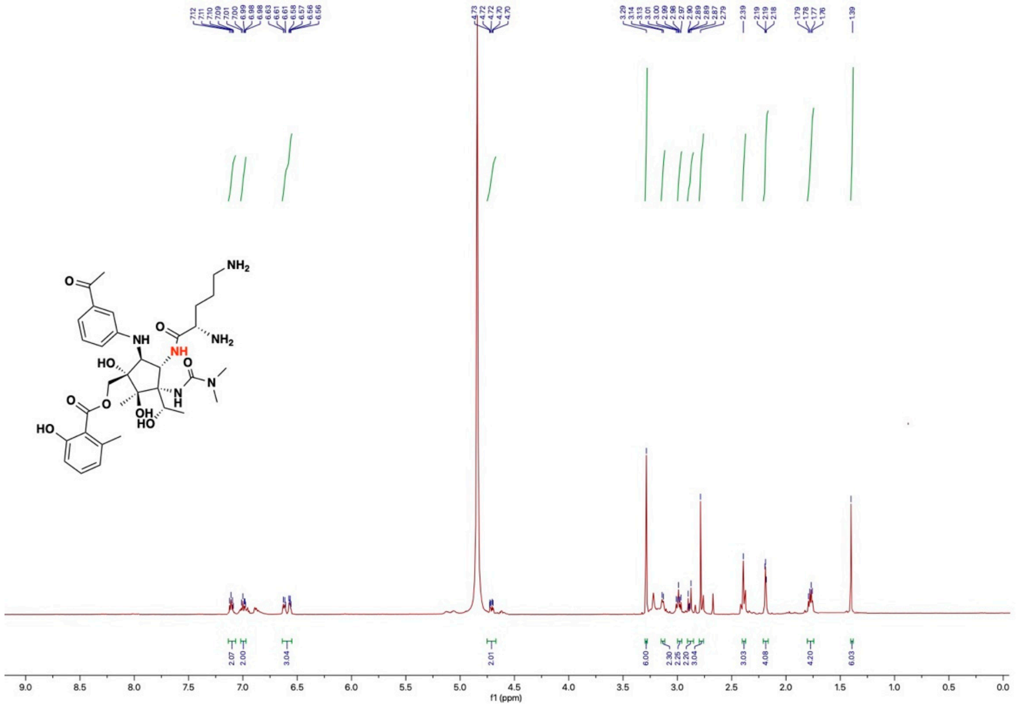

Figure S13. ^{1}H-NMR spectrum of compound D2

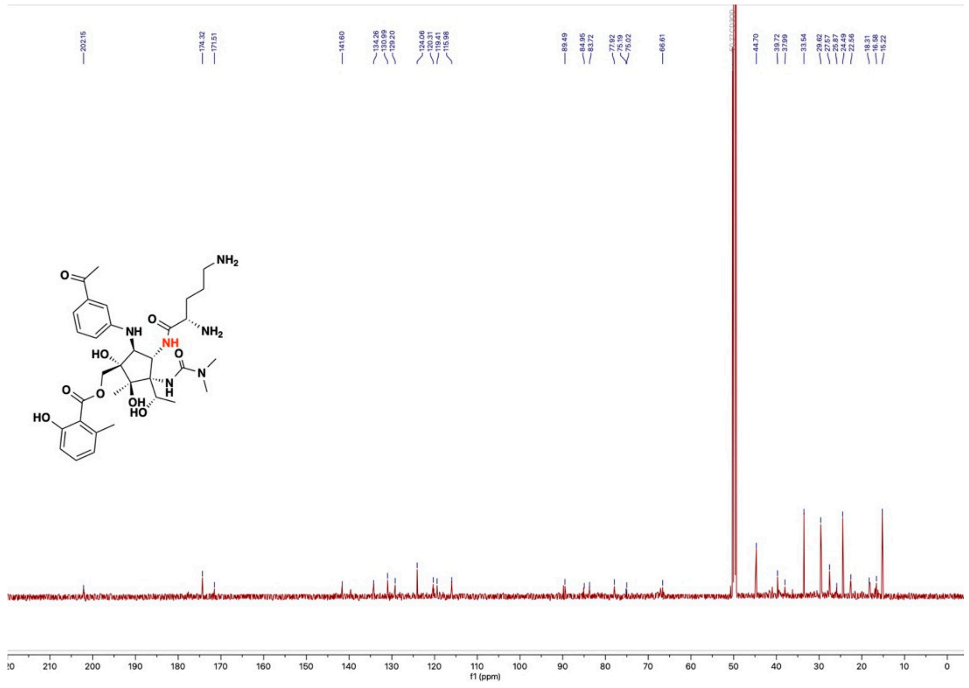

Figure S14. ^{13}C-NMR spectrum of compound D2

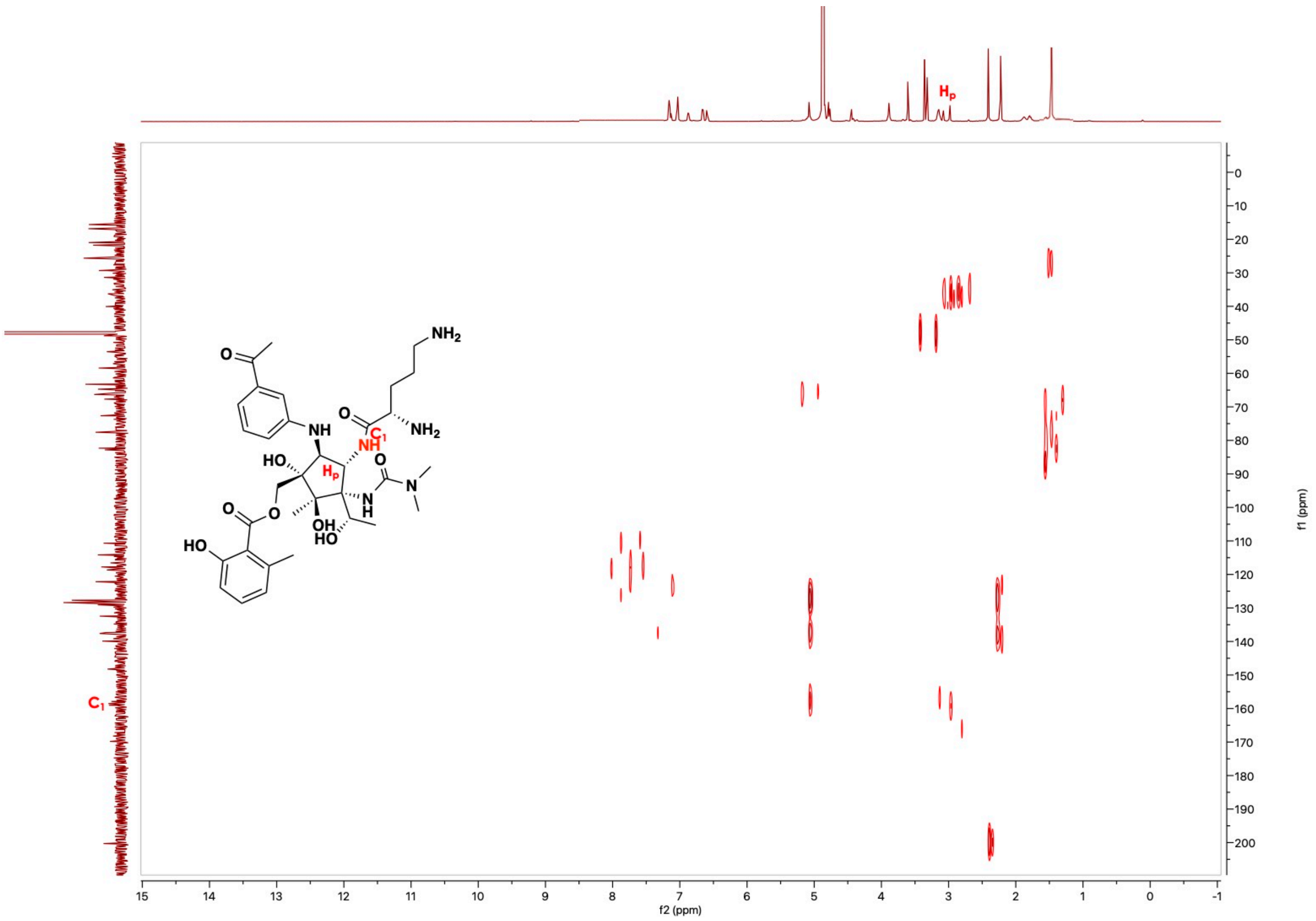

Figure S15. HMBC-NMR spectrum of compound D2

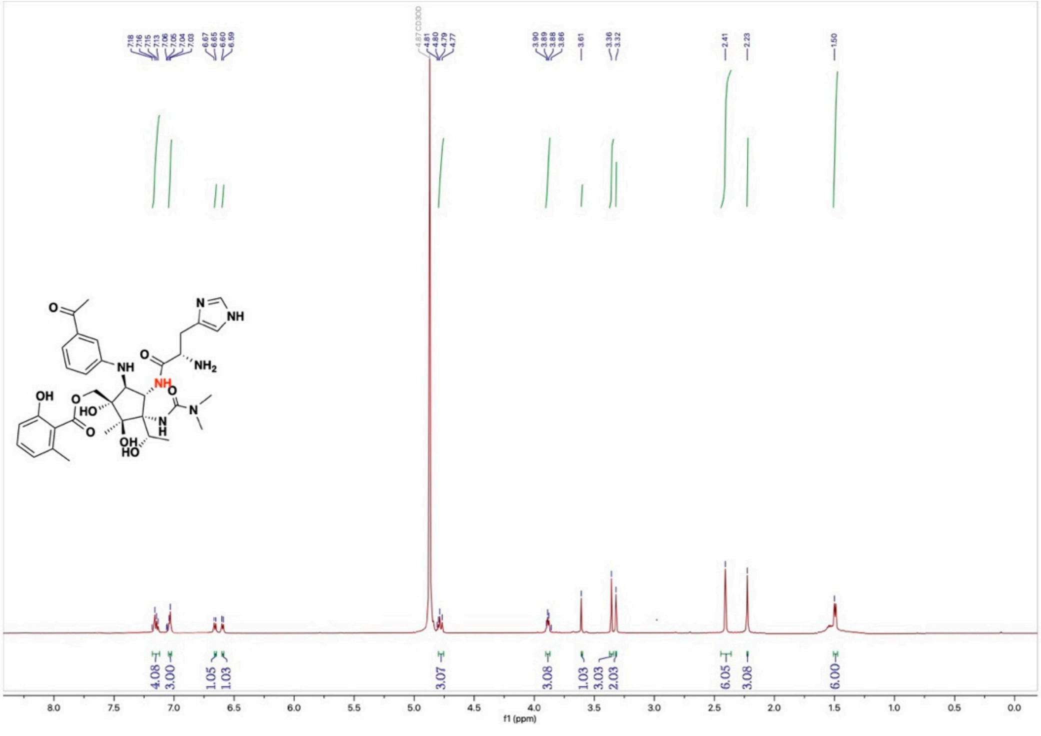

Figure S16. ^{1}H-NMR spectrum of compound D3

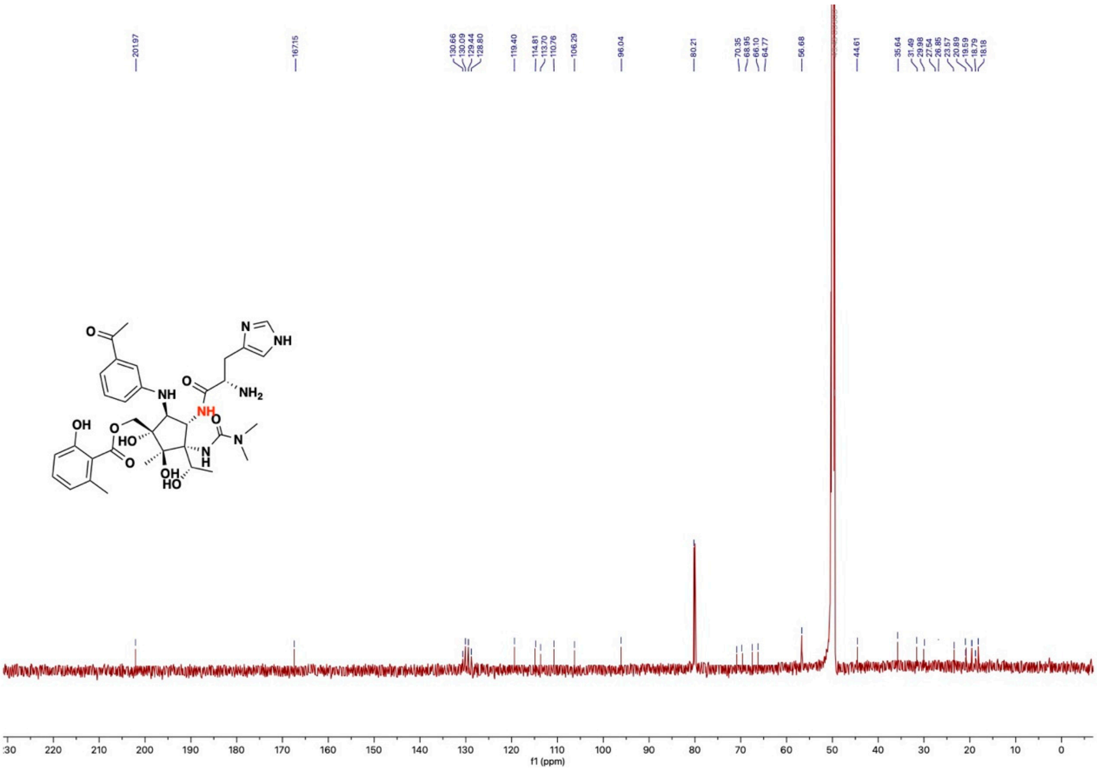

Figure S17. ^{13}C-NMR spectrum of compound D3

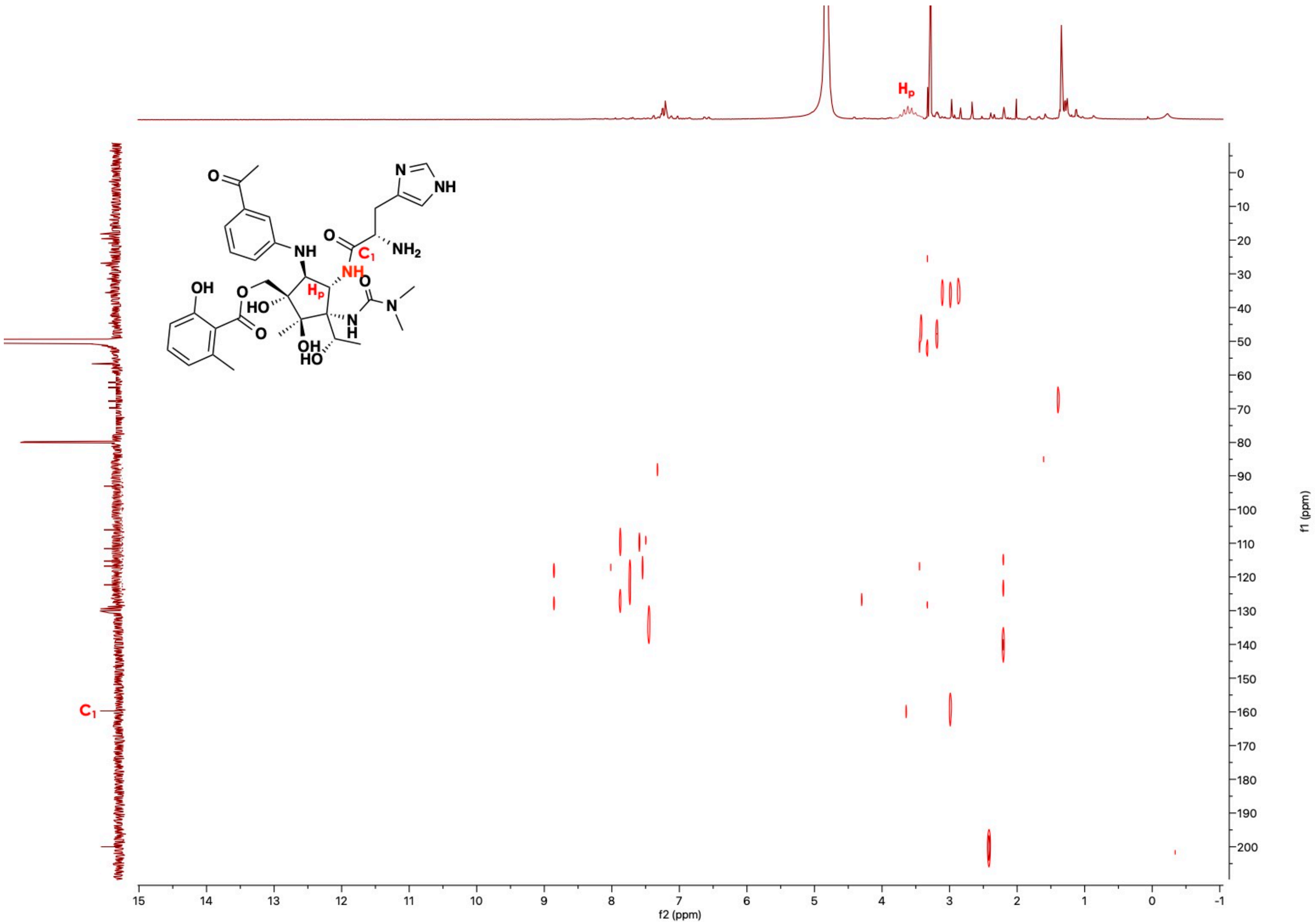

Figure S18. HMBC-NMR spectrum of compound D3
